# Regulation of respiratory syncytial virus nucleoprotein oligomerization by phosphorylation

**DOI:** 10.1016/j.jbc.2025.108256

**Published:** 2025-02-03

**Authors:** Vincent Basse, Yao Wang, Carine Rodrigues-Machado, Céline Henry, Charles-Adrien Richard, Cédric Leyrat, Marie Galloux

**Affiliations:** 1 Unité de Virologie et Immunologie Moléculaires (VIM), Université Paris-Saclay, INRAE, Jouy-en-Josas, France; 2 Institut de Génomique Fonctionnelle, Université de Montpellier, CNRS, INSERM, Montpellier, France; 3 Institut Micalis, Université Paris-Saclay, INRAE, AgroParisTech, Jouy-en-Josas, France

**Keywords:** RSV, nucleoprotein, phosphorylation, RNA encapsidation, viral factories

## Abstract

The negative-sense RNA genome of respiratory syncytial virus (RSV) is encapsidated by the viral nucleoprotein N, forming a left-handed helical nucleocapsid which serves as template for the viral polymerase. Specific oligomerization of N along the viral genome necessitates a switch of conformation of N, from the neosynthesized monomeric and RNA-free N protein, named N^0^, to N-RNA oligomers. Although the binding of the N-terminal part of RSV phosphoprotein P plays the role of chaperone to impair RNA binding to N, N^0^-P interaction alone is not sufficient to prevent N oligomerization. Here, we explored the potential role of post translational modifications that could participate in the stability of N^0^. Among the post translational modifications specifically identified on recombinant monomeric N, we validated the presence of a phosphorylation site on residue Y88 of N which modulates N oligomerization. Our results suggest that RSV N oligomerization depends on the regulation by post translational modifications.

The genome of *Mononegavirales* (*MNVs*) is a nonsegmented negative-sense RNA (1, 2), which is constitutively encapsidated by a nucleoprotein N, forming a helical nucleocapsid (NC). Genome encapsidation prevents its degradation and recognition by cellular RNases and sensors of the innate immune response, respectively. This NC is also the matrix for the viral polymerase L for both viral transcription and replication, thus playing a key role in viruses’ life cycle. Among *MNVs*, the respiratory syncytial virus (RSV), prototype of the *Pneumovirida*e family (1, 2), is the most common cause of acute lower respiratory infections in young children worldwide, responsible for bronchiolitis (3, 4) but also of severe respiratory infections in immunocompromised and elderly people (5, 6). Of note, the bovine RSV is also responsible for infection of calves with symptoms similar to those of infants, and has important economic impact in cattle (7, 8). The first vaccines to prevent severe human RSV disease in the elderly and pregnant women were approved by the Food and Drug Administration only in 2023 (GSK, Pfizer) (9, 10). For children, the only specific treatment commercialized for newborns remains humanized monoclonal antibodies directed against the fusion protein F responsible for viral entry: palivizumab, Synagis (11, 12); nirsevimab, Beyfortus (13, 14). Although recent huge progresses were made in the development of preventive treatments, those are expected to induce only a short-term protection, and efficient therapeutic treatments are still missing.

RSV genome, of 15.2 kb, displays 10 genes encoding for 11 viral proteins (15). RSV NCs form flexible left-handed helices with a noncanonical arrangement (16, 17). The N protein, 391 residues long, is composed of two globular domains (N_NTD_ and N_CTD_) separated by a flexible hinge region (18). The interface between N_NTD_ and N_CTD_ forms the RNA binding groove. The N protein also possesses two N and C terminal extensions (N- and C-arms) involved in N oligomerization: the N-arm of the N_i_ protomer binds to the N_i-1_ protomer, whereas the C-arm of N_i_ binds to the top of the N_CTD_ of the N_i+1_ protomer. These N arms were recently shown to play a critical role in the noncanonical arrangement of N protomers, which is responsible for the variations in RNA accessibility within NCs (17). The recognition of the NC by the viral polymerase L requires the presence of its cofactor, the phosphoprotein P which interacts with both L and NCs (19, 20, 21, 22). During viral transcription, the L polymerase sequentially synthesizes viral mRNAs and is responsible for 5′ end capping and methylation and 3′ polyadenylation of these mRNAs. Of note, RSV transcription also depends on the presence of the viral transcription factor M2-1, which improves the processivity of L along the full genome (23) and was shown to interact with both P and viral mRNAs (24, 25, 26, 27). On the contrary, genome replication only requires N, L, and P proteins, and leads to the synthesis of positive sense RNA antigenomes, serving as template for subsequent synthesis of viral genomes (28). Concomitantly to their synthesis, both antigenomes and genomes are encapsidated by N. Therefore, the presence of a pool of monomeric RNA-free N protein (named N^0^), available for encapsidation of antigenomes and genomes, constitutes a prerequisite for efficient viral replication. The viral N protein showcasing a high propensity to interact with RNA and to oligomerize, the regulation of a N^0^ pool and of the switch from N^0^ to N-viral RNA complex are critical for RSV replication. To stabilize the N^0^ form, all the *MNV*s share a conserved mechanism that relies on the P protein (or related viral protein) which acts as a chaperone of neosynthesized N (29, 30, 31). The RSV P protein thus plays a pivotal role in L activity, by acting as a hub between L, NC, M2-1, and N^0^ proteins, favoring the spatial proximity required for genome encapsidation. This protein is a tetramer with a central oligomerization domain flanked by intrinsically disordered regions (32). These N- and C-terminal intrinsically disordered regions allow the P protein to interact with N^0^ and M2-1 (33, 34), and with NC and L (19, 20, 21, 22), respectively. More specifically, the crystal structure of *MNV* N^0^-P complexes revealed that the N terminal part of P binds on N surface, competing with RNA binding and/or oligomerization, depending on the N^0^-P complex considered (35, 36, 37, 38, 39, 40). For RSV, although no crystal structure is available, we have shown that the stabilization of N^0^ not only depends on P, which induces a rotation of N_NTD_ relative to N_CTD_ compared to the oligomeric form, but also on a stacking of the C-terminal arm of N into the positively charged RNA groove, that blocks RNA binding (41), as also observed for the closely related human metapneumovirus (HMPV) N^0^-P complex (39). Furthermore, like the majority of *MNVs*, RSV transcription and replication are cytoplasmic, taking place within viral factories which are virus-induced organelles called inclusion bodies (IBs) (42, 43). The formation of these organelles, which were shown to depend on N-P interactions (44, 45), allows to isolate the viral and cellular proteins involved in viral RNA synthesis, thus participating in the encapsidation specificity of viral genome and antigenome. Although recent progress in the structural characterization of RSV N protein and its interactions with P has been made, polymerase functioning depends on a fine tuning of these transient protein-protein interactions which remain poorly understood.

In the present study, we investigated the potential role of post translational modifications (PTMs) of N that could improve the stability of the monomeric form, and that could be involved in the N^0^ to N-RNA transition. Using a fusion protein between the full-length N and the peptide derived from the first 40 N-terminal residues of P (P40), which was previously shown to mimic monomeric N^0^, we identified by mass spectrometry (MS) several PTMs of N. In particular, we identified the residue Y88 as a novel phosphorylated residue of the RSV N protein. By combining biochemical and cellular assays together with molecular dynamics (MD) simulations, our results suggest that phosphorylation of residue Y88 of N could stabilize the N monomeric form by favoring its interaction with the residue R27 which plays a key role in N oligomerization. Our results thus highlight an additional mechanism of control involved in the switch from RSV N^0^ to N-RNA.

## Results

### Identification of PTMs on the monomeric recombinant N-P40 protein

We have previously shown that the production in *Escherichia coli* of a recombinant fusion protein corresponding to the 40 N-terminal residues of P fused at the C terminus of full-length N protein (N-P40 protein) allows to purify a monomeric RNA-free protein. However, this protein still presented a high propensity to oligomerize and to interact with RNA (45). Here, we first wondered if its production in eukaryotic cells, by allowing specific PTMs compared to *E. coli*, could favor the stability of monomeric N-P40. The recombinant N-P40 protein, with a N-terminal 6xHis tag, followed by a tobacco etch virus (TEV) cleavage site, was thus produced in insect cells and purified using a protocol similar to that previously used (45).

**Figure 1:**
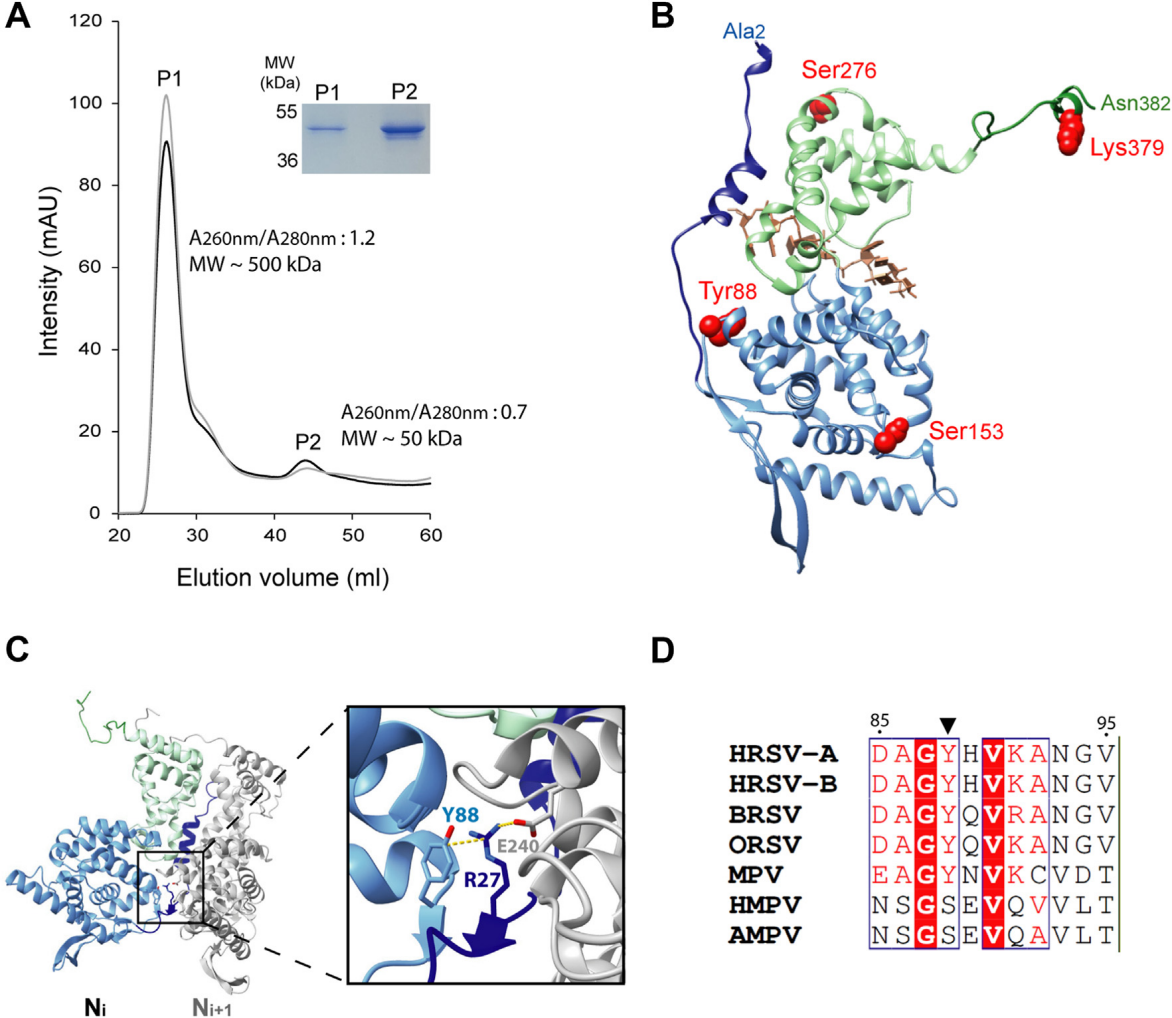
**Identification of PTMs on recombinant N-P40 protein.***A*, gel filtration profile of purified N-P40 produced in insect cells. The curves corresponding to absorbance at 260 nm and 280 nm are presented as *gray* and *black lines*, respectively. P1 and P2 indicate the two peaks detected. For each peak, *A*_260nm_/*A*_280nm_ ratio and apparent mass are indicated. The fractions corresponding to P2 were pooled, concentrated, and the sample was analyzed together with the sample of P1 by SDS-PAGE colored with Coomassie blue. *B*, 3D ribbon representation of the RSV N protein (PDB accession number 2WJ8) colored according to domains: the N- and C-arms are in *dark blue* and *green*, and the N_NTD_ and N_CTD_ are in light *blue* and *green*, respectively. The first and last residues (Ala2 and Asn382) observed on the crystal structure are indicated. Residues Y88, S153, S276, and K379 are represented by *red spheres*. *C*, structure of an N dimer (*left*) with the protomer N_i_ colored as in (*B*) and protomer N_i+1_ in *gray*. *Right*: close-up view of the interaction between the residues Y88 and R27 of the protomer N_i_, and between residue R27 of N_i_ protomer and E240 of N_i+1_ protomer. N residues are shown as *sticks*, and the interactions are represented by *yellow lines*. *D*, multiple sequence alignments of N sequences from *Pneumoviridae*. Invariant residues are highlighted in *white font* on a *red* background, and partially conserved residues are in *red*. The residue Y88 is indicated with an *arrow*. Abbreviations and UniProt accession codes: HMPV (human metapneumovirus, NCAP_HMPVC), AMPV (avian metapneumovirus, NCAP_AMPV1), ORSV (ovine respiratory syncytial virus, NCAP_ORSVW), BRSV-A (bovine RSV type A, NCAP_BRSVA), MPV (murine pneumonia virus, NCAP_MPV15), HRSV-A (human RSV type A, NCAP_HRSVA), HRSV-B (human RSV type B, NCAP_HRSVB). PDB, Protein Data Bank; PTM, post translational modifications; RSV, respiratory syncytial virus.

Unfortunately, gel filtration profile of purified N-P40 was similar to the one obtained for the protein produced in *E. coli*, with two peaks: a major peak P1 corresponding to N-P40-RNA oligomers (*A*_260nm_/*A*_280nm_ ratio ˃ 1 and apparent mass of ∼500 kDa, estimated from the Superdex 200 calibration), and a minor peak P2 corresponding to N-P40 RNA-free monomers (*A*_260nm_/*A*_280nm_ ratio <1, and apparent mass of ∼ 50 kDa) (Fig. 1*A*). Of note, analysis of P1 and P2 samples by SDS-PAGE stained with Coomassie blue showed the presence of a single band with a molecular weight close to 50 kDa in both peaks (Fig. 1*A*), consistent with the expected size of the N-P40 protein. The fractions of monomeric N-P40 were pooled and concentrated, and analysis of PTMs by mass spectrometry (liquid chromatography with tandem mass spectrometry [LC-MS/MS]) was performed on N-P40 isolated from P1 and P2 peaks. Some peptides carrying PTMs were detected by LC-MS/MS analysis, proving the presence of PTMs on both the oligomeric and monomeric protein, with several being found on both forms of N-P40. Of note, peptides corresponding to the TEV cleavage site (ENLYFQS) with the tyrosine residue phosphorylated were also detected. We decided to focus on PTMs specifically identified on the monomeric N-P40. Analysis of the signals detected by LC-MS/MS allowed to identify three phosphorylation sites on residues Y88, S153, and S276, and a di-methylation of residue K379 (Table 1, and Fig. S1). The LC-MS/MS signals being of low intensity, we first evaluated the potential impact of the modification of these residues on N, based on its structure and the data from the literature. The residue Y88 is located at the end of the α-helical domain of the N_NTD_ and is involved in the interaction between the N-arm and N_NTD_ (17, 18), whereas the S153 is exposed to the solvent at the surface of N_NTD,_ close to the binding site of the P C terminus on NCs (20) (Fig. 1*B*). The residue S276 is at the surface of the N_CTD_, at the proximity of the P40 binding site based on the model of RSV N^0^-P complex (41). Finally, the K379 residue is located in the C terminal arm of N, which was shown to play a key role in the orientation of N protomer within NCs (17), but also in the inhibition of RNA binding in the N^0^-P conformation (41). Based on their localization, most of these PTMs could impact the oligomerization status of N. However, the only PTM detected in parallel on peptides derived from the recombinant N-P40 produced in *E. coli*, although with relatively low-quality scores, was the phosphorylation of Y88 residue (Table 1). Due to the similar gelfiltration profiles of recombinant N-P40 purified from eukaryotic cells or *E. coli*, we hypothesized that Y88 phosphorylation may be the main PTM responsible for the stability of the monomeric form.

**Table 1:** Modified peptides identified by LC-MS/MS

N-P40 production	PTM^a^	Peptides sequence	−10logP (1–100)^b^
Insect cells	Phosphorylation	DAG**Y**_**88**_HVKANGV	24.61
	Phosphorylation	EMGEVAPEYRHD**S**_**153**_PDCGMIILCIAALVITK	28.79
	Phosphorylation	NIMLGHA**S**_**276**_VQAEMEQVVEVYEYAQK	17.93–40.99
	Di-methylation	DLTAEELEAI**K**_**379**_HQLNPK	36.01
*Escherichia coli*	Phosphorylation	DAG**Y**_**88**_HVKANGVDVTTHR	19.81

Residues with a PTM are indicated in bold.

a Mass weight gain: Phosphorylation (+79.97), di-methylation +28.03.

b Peaks software score (−10logP).

Overall, our results allowed to identify PTMs specific to the monomeric N, and suggest that phosphorylation of residue Y88 of N could participate in the stabilization of the monomer. Noteworthy, Y88 is involved in a key interaction with the residue R27 which is critical for N oligomerization (Fig. 1*C*) (18, 46). Finally, multiple sequence alignment of *Pneumoviridae* N proteins reveals that Y88 is conserved for all the orthopneumoviruses and corresponds to a Serine for metapneumoviruses (Fig. 1*D*), which could also be phosphorylated.

### The residue Y88 of N is critical for viral polymerase activity and the formation of viral factories

**Figure 2:**
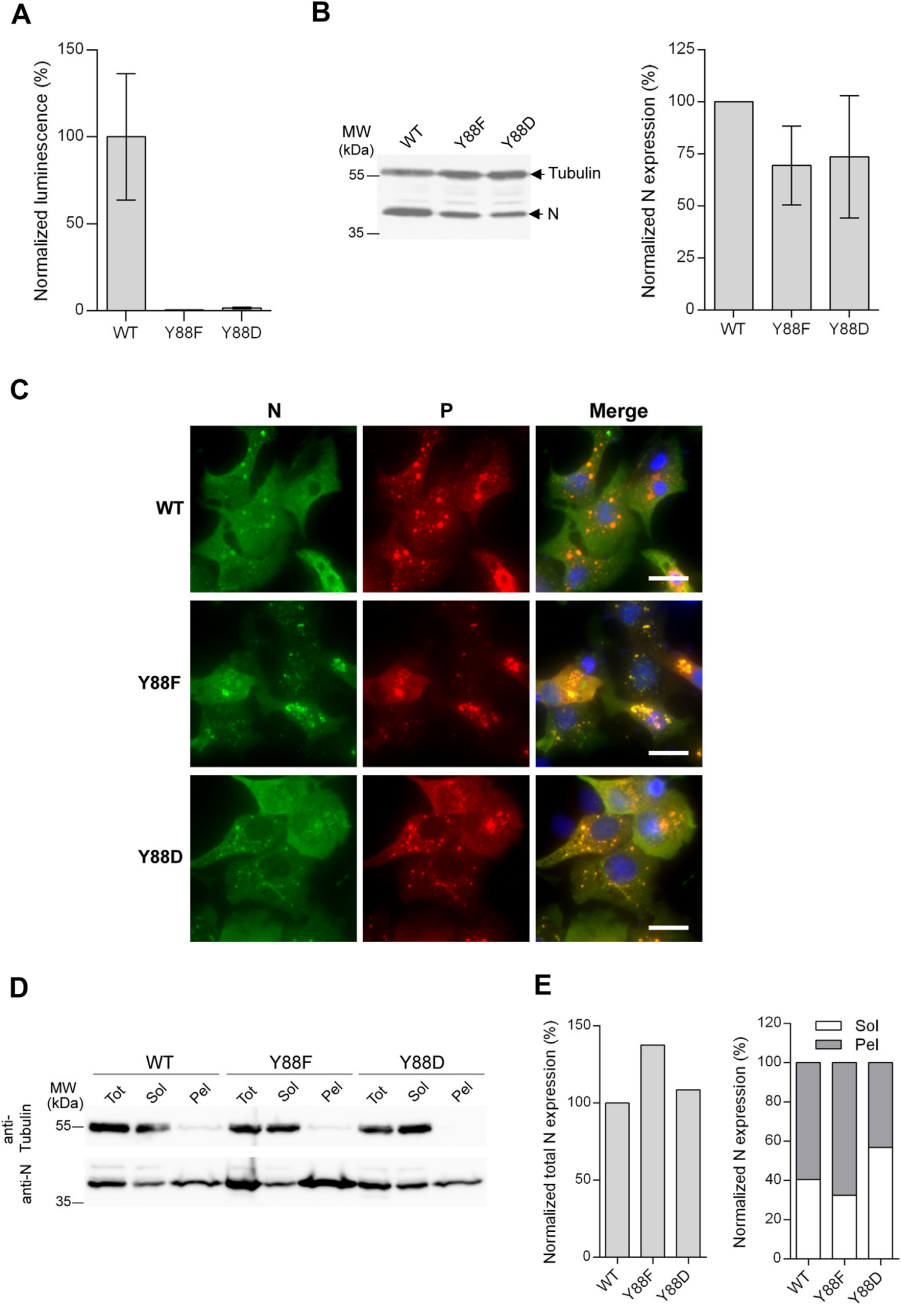
**Mutations of Y88 of N affect RSV polymerase activity and IBs formation.***A*, BSRT7/5 cells were transfected with plasmids encoding the WT P, M2-1, and L proteins, the pMT/Luc minigenome, and WT or Y88 mutants of the N protein, together with pCMV-βGal for transfection standardization. Cells were lysed 24 h post transfection and viral RNA synthesis was quantified by measuring the luciferase activity. Each luciferase minigenome activity value was normalized based on β-galactosidase expression. The graph corresponds to one representative experiment out of three independent experiments performed in quadruplicate. Error bars represent standard deviations (±SD) calculated based on one representative experiment. *B*, representative Western blot showing the expression of N protein variants in BSRT7/5 cells (*left*), and quantification of N expression from two independent Western blots (*right*). Signals of N were normalized based on the intensity of tubulin signals and of the values obtained for WT N expression. *C* and *D*, BSRT7/5 cells were transfected with plasmids encoding N (WT or mutants) and P. *C*, cells were fixed 24 h post transfection and labeled with anti-P (*red*) and anti-N (*green*) antibodies, and the distribution of viral proteins was observed by fluorescence microscopy. Nuclei were stained with Hoechst 33342. The bars represent 20 μm. *D*, cells were lyzed 24 h post transfection and the proportion of N in the total (Tot), soluble (Sol), and pellet (Pel) fractions was analyzed by Western blot. Tubulin was used as a control. *E*, quantification of the level of N WT and Y88 mutants expression from (*D*) (*left*), and of the proportion of N in the pellet (Pel) and the soluble (Sol) fractions in each condition, normalized based on the total (Tot) signal intensity determined for each protein (*right*). All quantifications were performed using ImageJ software. IBs, inclusion bodies; RSV, respiratory syncytial virus.

In order to investigate the importance of residue Y88 and of its potential phosphorylation for N activity, we first attempted to determine the impact of mutations of this residue on the viral polymerase activity using the minigenome assay, as described previously (20, 24). We chose to substitute Y88 by either a phenylalanine or an aspartic acid, which are supposed to mimic a similar lateral chain steric hindrance or a phosphorylation, respectively. Both substitutions Y88F and Y88D totally abrogated RSV polymerase activity (Fig. 2*A*). Although these two mutations seemed to slightly affect the production or stability of N (Fig. 2*B*), such difference cannot explain the total inhibition of the L activity, compared to data from previous studies (20, 41). To further assess the impact of these mutations, we then studied the cellular localization of N. As previously described, coproduction of WT N and P proteins leads to the formation of spherical cytoplasmic organelles where N and P colocalize, validating the phase separation responsible for the formation of pseudo IBs (42, 43, 47) (Fig. 2*C*), whereas coproduction of a mutated monomeric N with P does not allow to induce the formation of these organelles (41, 48). As shown on Figure 2*C*, expression of the Y88F mutant of N led to the formation of cytoplasmic non spherical aggregates where both N and P colocalize. On the contrary, mutation Y88D of N led to the formation of numerous pseudo-IBs of small size compared to the control condition (Fig. 2*C*), revealing a defect of the phase separation capacity upon mutation of N. We also analyzed the solubility of WT and mutant N proteins when expressed alone in cells, by quantification of the protein in the cell lysate and the pellet. WT N was present in both the soluble and the pellet fractions (Fig. 2, *D* and *E*), as previously observed (48). In comparison, the mutation Y88F was shown to increase the level of N production and to induce its accumulation in the pellet fraction (Fig. 2, *D* and *E*). Although we cannot exclude that the accumulation of N Y88F in the pellet could be due to the higher level of expression of this mutant compared to the WT, this observation correlates with the presence of aggregates of N in the cytoplasm of transfected cells (Fig. 2*C*), and suggests that this mutation might mainly induce aberrant N oligomerization. However, we also observed a slight difference of solubility for the mutant Y88D compared to the WT N, with more N protein in the soluble fraction (Fig. 2, *D* and *E*). Of note, the tubulin used as a control was recovered only in the soluble fraction.

These data support the importance of the residue Y88 of N for the polymerase activity and its role in N folding. Furthermore, the impact of Y88D mutation on pseudo-IBs formation and N solubility suggests that mimicking Y88 phosphorylation could stabilize the monomeric form of N.

### The mutation Y88D stabilizes the monomeric form of recombinant N-P40 protein

**Figure 3:**
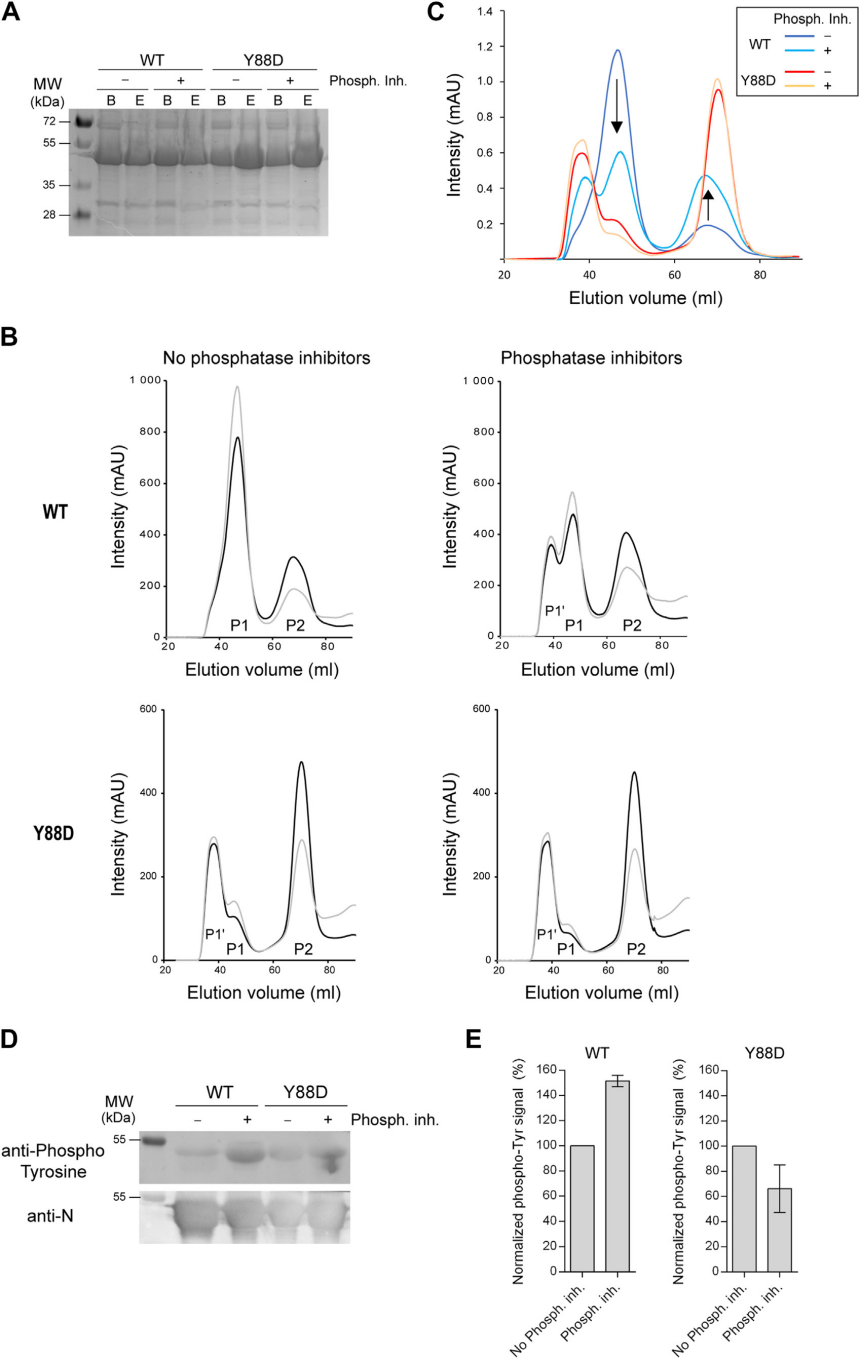
**Impact of phosphatase inhibitors and Y88D mutation on purified recombinant N-P40 proteins produced in *Escherichia coli*.***A*, SDS-PAGE and Coomassie blue staining of proteins on beads before elution (*B*) and of eluted N-P40 proteins (*E*). *B*, gel filtration profiles of purified N-P40 WT (*upper panels*) and Y88D mutant (*lower panels*) produced in *E. coli*, purified in the absence (*left panels*) or the presence of phosphatase inhibitors (*right panels*). The curves corresponding to the absorbance at 260 nm and 280 nm are presented as *gray* and *black lines*, respectively. P1, P1′ indicate the peaks of N-P40-RNA oligomers, and P2 the peak of monomers. *C*, overlay of the four normalized curves of gel filtration profiles at 280 nm, after baseline correction and area integration. *Black arrows* indicate the decrease of amplitude of the N-P40-RNA oligomers peak, and the increase of monomeric N-P40 peak, respectively. *D*, representative Western blot analysis of monomeric WT and Y88D N-P40 proteins (from peak P2) purified in the absence or the presence of phosphatase inhibitors using anti-phosphotyrosine and anti-N antibodies. *E*, quantification of the level of phosphotyrosine signals from two independent Western blots, normalized based on the signals obtained with anti-N antibody, and the values of intensities for each N purified in the absence of phosphatase inhibitors.

We then studied the impact of Y88D mutation on the recombinant N-P40 protein compared to WT protein produced in *E. coli*, which was already characterized in a recent study (45), in the presence or the absence of phosphatase inhibitors. In that case, we also decided to improve the protocol of purification by increasing the strength of the washes compared to our initial protocol of purification, with two washes in the presence of 100 mM imidazole, and by incubating the samples in the presence of RNase A overnight before elution, in the presence or absence of phosphatase inhibitors. SDS-PAGE analysis of the beads before elution and of the samples after elution revealed that the quantity of protein was similar in all the samples (Fig. 3*A*), suggesting that the mutation Y88D did not affect N-P40 production, nor that addition of phosphatase inhibitors modified the levels of purified proteins. As shown on the gel filtration profiles of WT N-P40, in the absence of phosphatase inhibitors, this protocol allowed to decrease the proportion of N-RNA oligomers (P1 peak) and to increase the level of the monomeric fraction (P2 peak) (Fig. 3*B*), compared to our previous results (45). More interestingly, purification of WT N-P40 in the presence of phosphatase inhibitors induced an increase of the proportion of the monomeric protein (Fig. 3, *B* and *C*). Of note, in the presence of phosphatase inhibitors, we also noticed the presence of a second peak of N-P40-RNA oligomers (P1′ peak), which may correspond to aggregates (Fig. 3, *B* and *C*). In comparison, in the absence of phosphatase inhibitors, a higher quantity of monomeric N-P40 was purified for the Y88D mutant compared to the WT N-P40 protein (Fig. 3, *B* and *C*), and only a low proportion of N-P40-RNA oligomers was detected. The elution volume of these oligomers is similar to the one of the P1′ peak observed for the WT N-P40-RNA oligomers purified in the presence of phosphatase inhibitors. These observations suggest that the Y88D mutation induced the aggregation of the protein at high concentration. However, no main difference of the gel filtration profile was observed for the mutant Y88D purified in the presence of phosphatase inhibitors. Finally, we analyzed the tyrosine phosphorylation status of these purified monomeric N-P40 proteins by Western blot, using an anti-phosphotyrosine antibody. As shown on Figure 3*D*, whereas purification in the presence of phosphatase inhibitors induced an increase of the signal of phosphorylated tyrosine for the WT protein, no significant difference of phosphorylation was observed for the mutated Y88D mutant between the two conditions of purification. These observations first suggest the presence of different phosphorylated tyrosine residues on recombinant N-P40. As previously mentioned, the phosphorylation of the tyrosine of the TEV cleavage site could participate to this signal. Noteworthy, these data also correlate with the results from Asenjo *et al.* (49), and the potential phosphorylation of different tyrosine residues of N.

Altogether, these results strongly support that phosphorylation might play a role in the stabilization of the monomeric form of N, and that the residue Y88 could be the main phosphorylation site involved in this mechanism.

### Mutation Y88D impairs RNA encapsidation and pseudo-IBs formation *in vitro*

**Figure 4:**
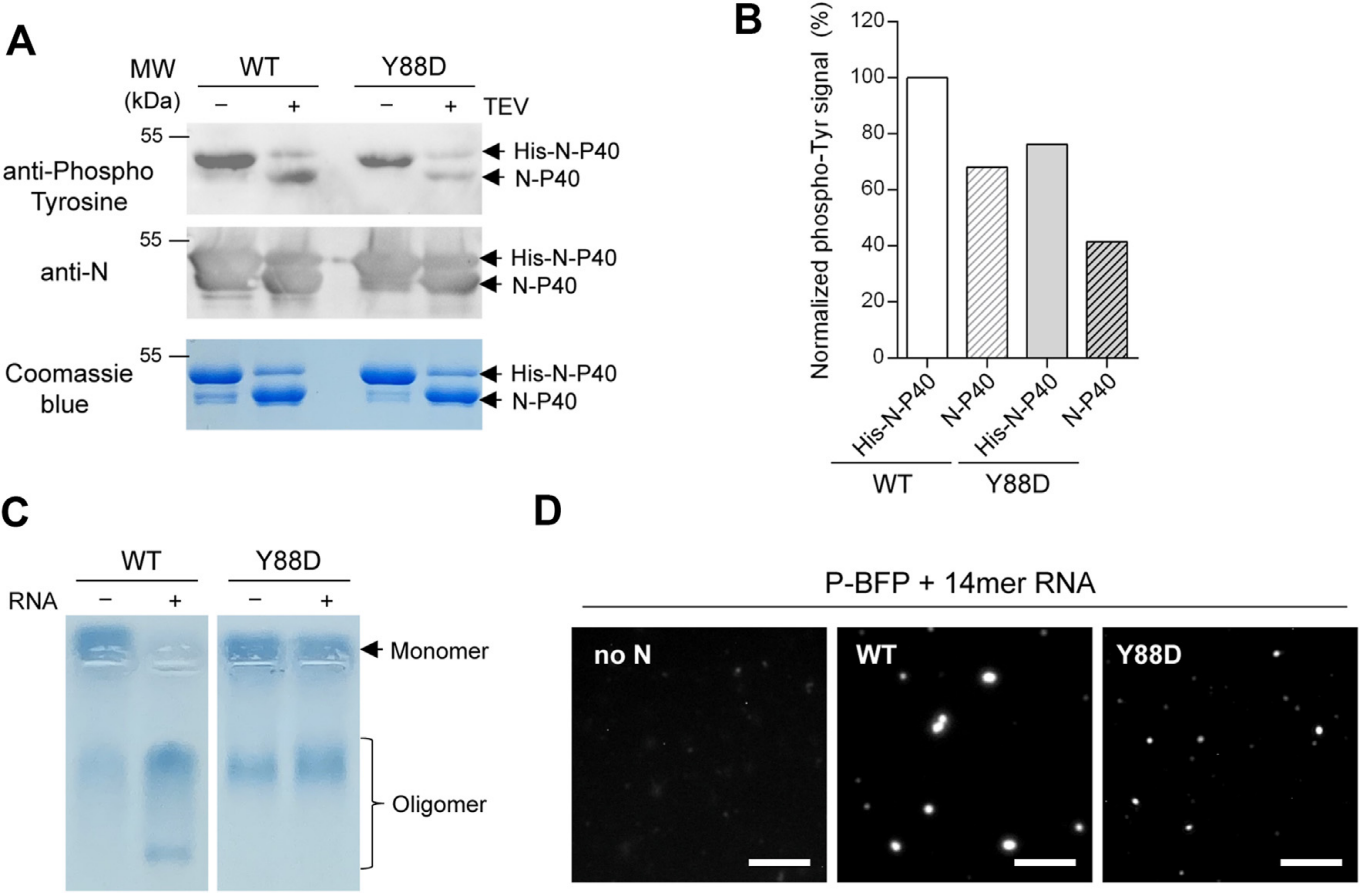
**Impact of Y88D mutation on RNA encapsidation and pseudo-IBs formation *in vitro*.***A*, Western blot (*upper panels*) and SDS-PAGE and Coomassie blue staining (*lower panel*) analysis of the purified monomeric N-P40 recombinant proteins before and after cleavage of the N-terminal 6xHis tag by TEV. For Western blot, membranes were incubated either in the presence of anti-N or with anti-phospho-tyrosine antibodies. The results presented are representative of two independent experiments. *B*, quantification of the signals corresponding to phosphorylated His-N-P40 and N-P40 proteins using ImageJ software. Phosphotyrosine signals were normalized to the corresponding N signals, and the normalized signal of WT His-N-P40 was used as reference (the 100% value) to determine the percentages of phosphorylation for each condition. *C*, analysis of recombinant N-P40 migration alone or incubated in the presence of 14-mer RNAs by agarose gel electrophoresis. Gels were stained with amido black. *D*, recombinant P-BFP protein (3.5 μM) with 14-mer RNAs (50 μM) were incubated alone or in the presence of monomeric N-P40 (WT or Y88D) proteins (13.5 μM) in the presence of 15% Ficoll. The formation of droplets was observed by fluorescence microscopy. The scale bar represents 10 μm. IBs, inclusion bodies; TEV, tobacco etch virus.

In order to further characterize the impact of Y88D substitution on N-P40 stability, we decided to perform experiments with the proteins purified in the absence of phosphatase inhibitors, to minimize the impact of other potential phosphorylation sites. As LC-MS/MS analysis revealed that the tyrosine residue of the TEV cleavage site could be phosphorylated, we first compared the phosphorylation status of purified N-P40 proteins before and after cleavage of the 6xHis tag by the TEV protease. As shown on the Figure 4*A*, similar quantity of full-length or cleaved proteins were observed between WT or Y88D mutant, as assessed by SDS-PAGE and Coomassie blue staining or by Western blot. Both full-length and cleaved proteins were revealed by Western blot using an anti-phosphotyrosine antibody, but the intensity of these signals was lower after the cleavage, validating that phosphorylation of the TEV cleavage site could participate to the signal. In both conditions, the intensity of the signals was quite lower for the Y88D mutant compared to those quantified for the WT protein (Fig. 4, *A* and *B*). Again, these observations suggest the presence of different phosphorylated tyrosine residues on recombinant N-P40, but also correlate with the phosphorylation of Y88 detected by LC-MS/MS. We then analyzed the potential impact of this mutation on the recombinant N-P40 stability by assessing its capacity to encapsidate RNA. The migration profiles on agarose native gel of monomeric N-P40 proteins alone or incubated in the presence of 14-mer RNA was first analyzed. For both WT and Y88D mutant proteins, we first observed the presence of oligomers in the absence of RNA (Fig. 4*C*). This suggested that these proteins still presented a tendency to aggregate upon concentration ˃1 mg/ml, and correlated with the presence of the P1′ peak on gel filtration profiles (Fig. 3, *B* and *C*). As previously observed (45), incubation of WT N-P40 induced a shift of protein migration from monomers to N-P40-RNA oligomers (Fig. 4*C*). However, a strong defect of RNA encapsidation was observed for the mutant Y88D, with most of the protein remaining monomeric. In order to confirm this result, the capacity of purified monomeric N-P40 proteins to form pseudo-IBs *in vitro* in the presence of fluorescent P-BFP and 14-mer RNAs, which depends on RNAs encapsidation (45), was assessed by fluorescence microscopy. As previously observed, coincubation of WT N-P40 with P-BFP and RNAs in the presence of 15% Ficoll led to the formation of small droplets of P (Fig. 4*D*), whereas fewer and smaller droplets were observed for Y88D mutant incubated with P-BFP, suggesting a defect of phase separation.

Altogether, our results strongly support that Y88 phosphorylation of N could stabilize the monomeric form.

### Impact of the mutation Y88D on the cellular localization of N-P40 coexpressed with P and N in cells

**Figure 5:**
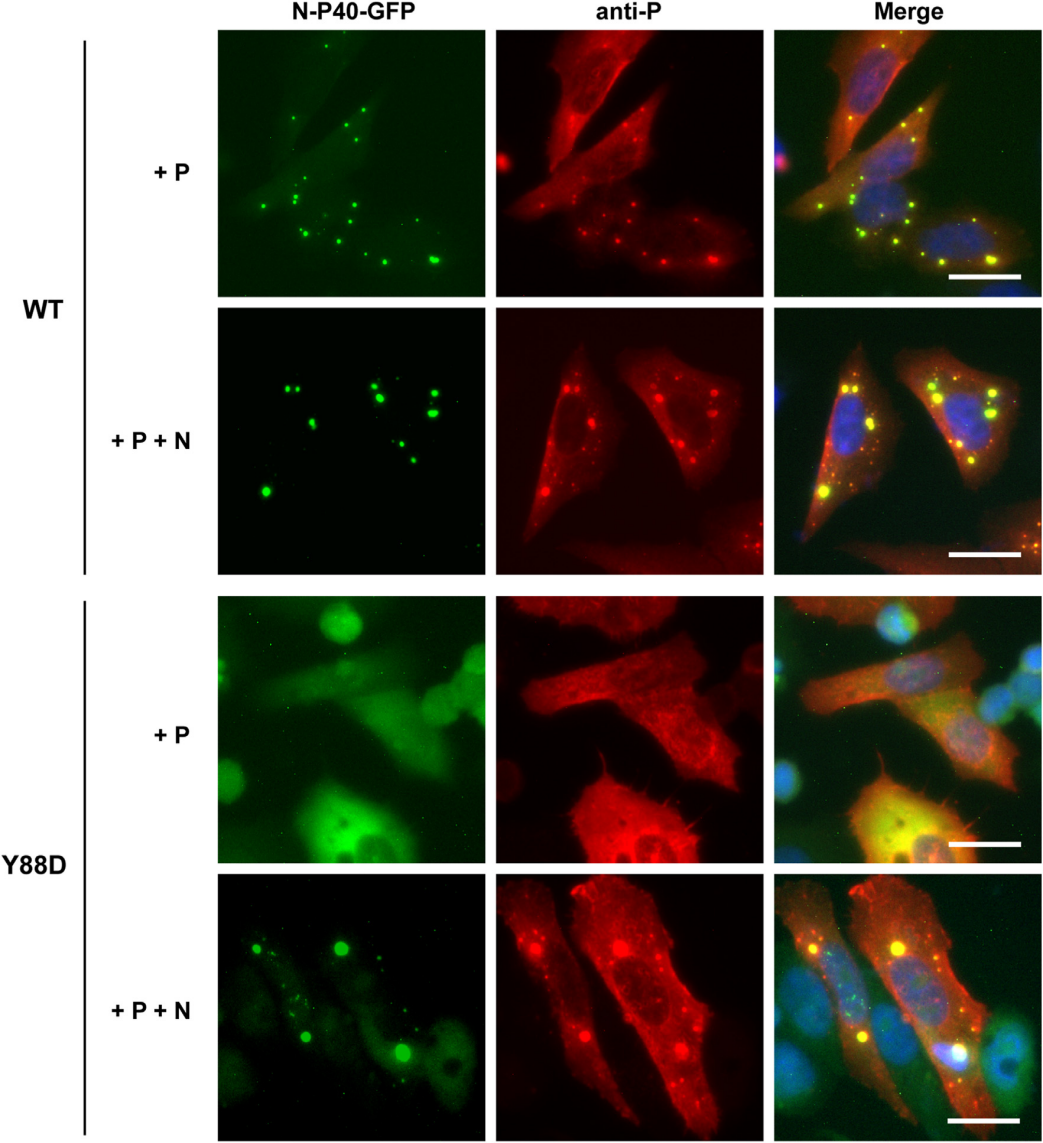
**Study of the cellular localization of N-P40-GFP protein.** HEp-2 cells were transfected with plasmids encoding N-P40-GFP (WT or Y88D mutant) and P alone or P and N proteins. Cells were fixed 24 h post transfection and labeled with anti-P (*red*) antibodies, and the distribution of N-P40-GFP and P proteins was observed by fluorescence microscopy. Nuclei were stained with Hoechst 33342. The scale bars represent 20 μm.

We finally assessed the capacity of N-P40 WT and Y88D mutant to form pseudo-IBs in eukaryotic cells. HEp-2 cells were cotransfected with plasmids encoding P and N-P40 fused to GFP at the C terminus (N-P40-GFP), in the absence or the presence of WT N protein. Coproduction of WT N-P40-GFP with P allowed to observe the formation of small spherical cytoplasmic inclusions where the two proteins colocalized, whereas both the mutant Y88D N-P40-GFP and P proteins presented a diffuse cytoplasmic localization in the same condition (Fig. 5). These observations correlated with *in vitro* data showing that WT N-P40 still presented the capacity to form pseudo IBs in the presence of RNA and P, while the mutation Y88D would stabilize the monomeric form, impairing the formation of pseudo-IBs. However, in the presence of N, pseudo-IBs similar to those observed upon coexpression of N and P alone were observed (Figs. 2*C* and 5), where both the WT and Y88D N-P40-GFP proteins were recruited. Such observation suggested that although Y88D mutation stabilized the monomeric N form, this monomeric N can be recruited within pseudo-IBs (Fig. 5).

These data thus validate the impact of Y88D mutation on pseudo-IBs formation in cells, suggesting that phosphorylation of Y88 could be critical for the stabilization of the monomeric form of N. More interestingly, the study of the localization of the mutant Y88D N-P40-GFP also allowed to reveal for the first time the recruitment of monomeric N to pseudo-IBs.

### Molecular dynamics simulation predicts that Y88 phosphorylation stabilizes N monomer

**Figure 6:**
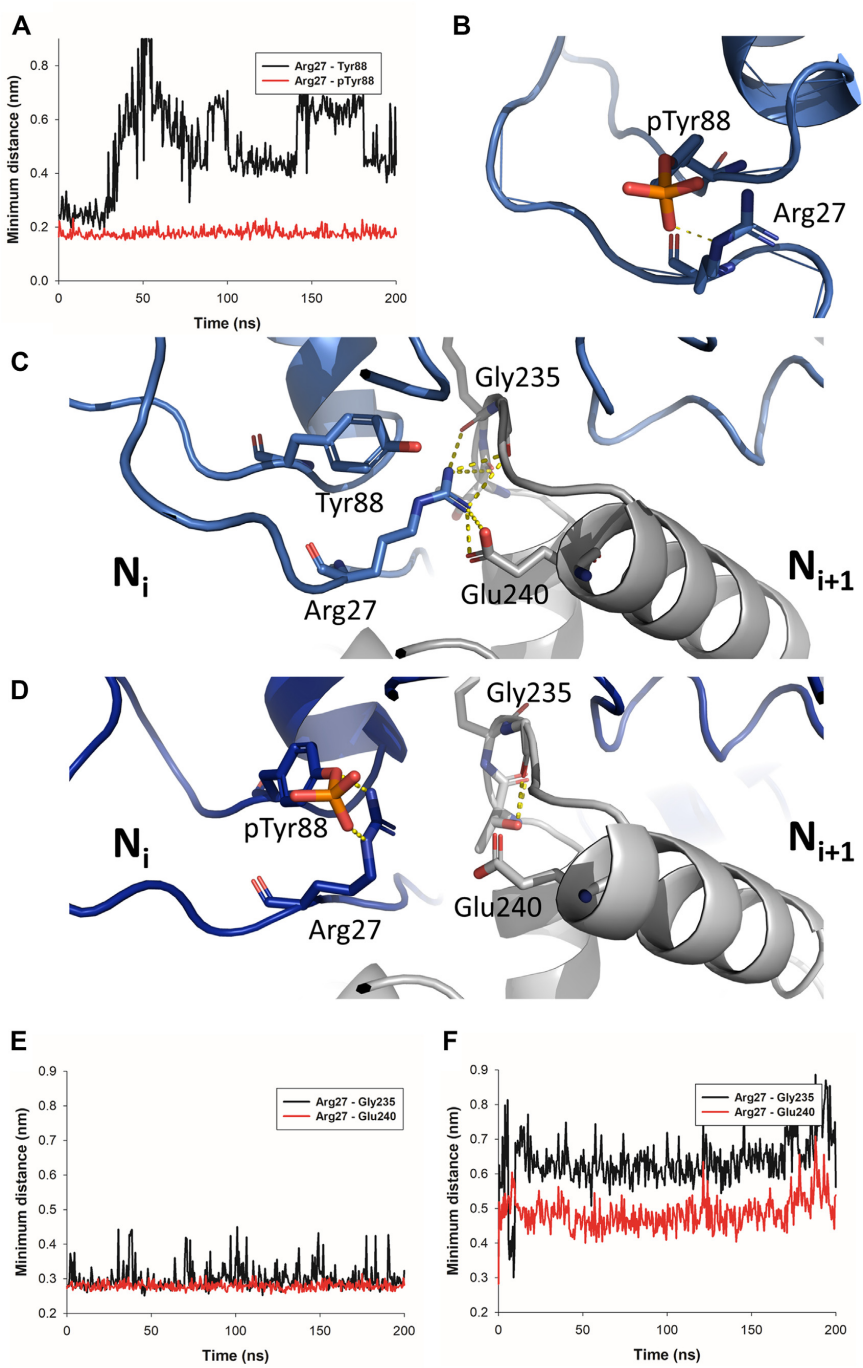
**Effect of Y88 phosphorylation on monomeric and dimeric N studied by molecular dynamics simulations.***A*, minimum distance as a function of simulation time between Arg27 and unphosphorylated Tyr88 (*black line*) or phosphorylated Tyr88 (*red line*) in monomeric N MD simulations. The distances were taken from representative MD trajectories performed in triplicates. *B*, close-up view of the Arg27/pTyr88 interaction at the end of the MD trajectory of monomeric N with phosphorylated Tyr88. *C*, close-up view of the Arg27/Tyr88 region at the interface between N_i_ and N_i+1_ protomers at the end of the MD trajectory shown in *panel A*. *D*, close-up view of the Arg27/pTyr88 region at the interface between N_i_ and N_i+1_ protomers at the end of the MD trajectory shown in *B*, illustrating the loss of contacts between Arg27 sidechain and the N_i+1_ protomer when Tyr88 is phosphorylated. *E*, minimum distance as a function of simulation time between Arg27 of N_i_ protomer and Gly235 (*black line*) or Glu240 (*red line*) of N_i+1_ protomer in the dimeric N model. The distances were taken from a representative MD trajectory performed in triplicates. *F*, same as *E* but in the presence of Tyr88 phosphorylation. MD, molecular dynamics.

In order to better understand the impact of Y88 phosphorylation on the N protein, we performed explicit solvent MD simulations of N in its monomeric and dimeric states (Fig. 6). In MD simulations of N monomer with phosphorylated Y88, the phosphate group interacts with the guanidinium group of R27 side chain, and this contact remains stable for the duration of simulation (Fig. 6, *A* and *B*). On the contrary, the distance between Y88 and R27 shows large fluctuations in the absence of phosphorylation (Fig. 6*A*), indicating that Y88 phosphorylation has a stabilizing effect on monomeric N. In experimental structures of oligomeric N-RNA complexes, Y88 is engaged in a cation-pi interaction with R27, as well as a network of hydrogen bonds involving G235 carbonyl oxygen and E240 carboxylate from the N_i+1_ protomer. These interprotomer interactions tend to remain stable in MD simulations of dimeric N, as shown in Figure 6, *C* and *E*. In contrast, when Y88 is phosphorylated, electrostatic interactions between the phosphate and R27 guanidinium group are formed, which tend to disfavor R27 contacts with G235 and E240 from the N_i+1_ protomer, as can be seen in Figure 6, *D* and *F*. Taken together, these results suggest that Y88 phosphorylation induces both a stabilizing effect on monomeric N and a destabilizing effect on oligomeric N.

## Discussion

**Figure 7:**
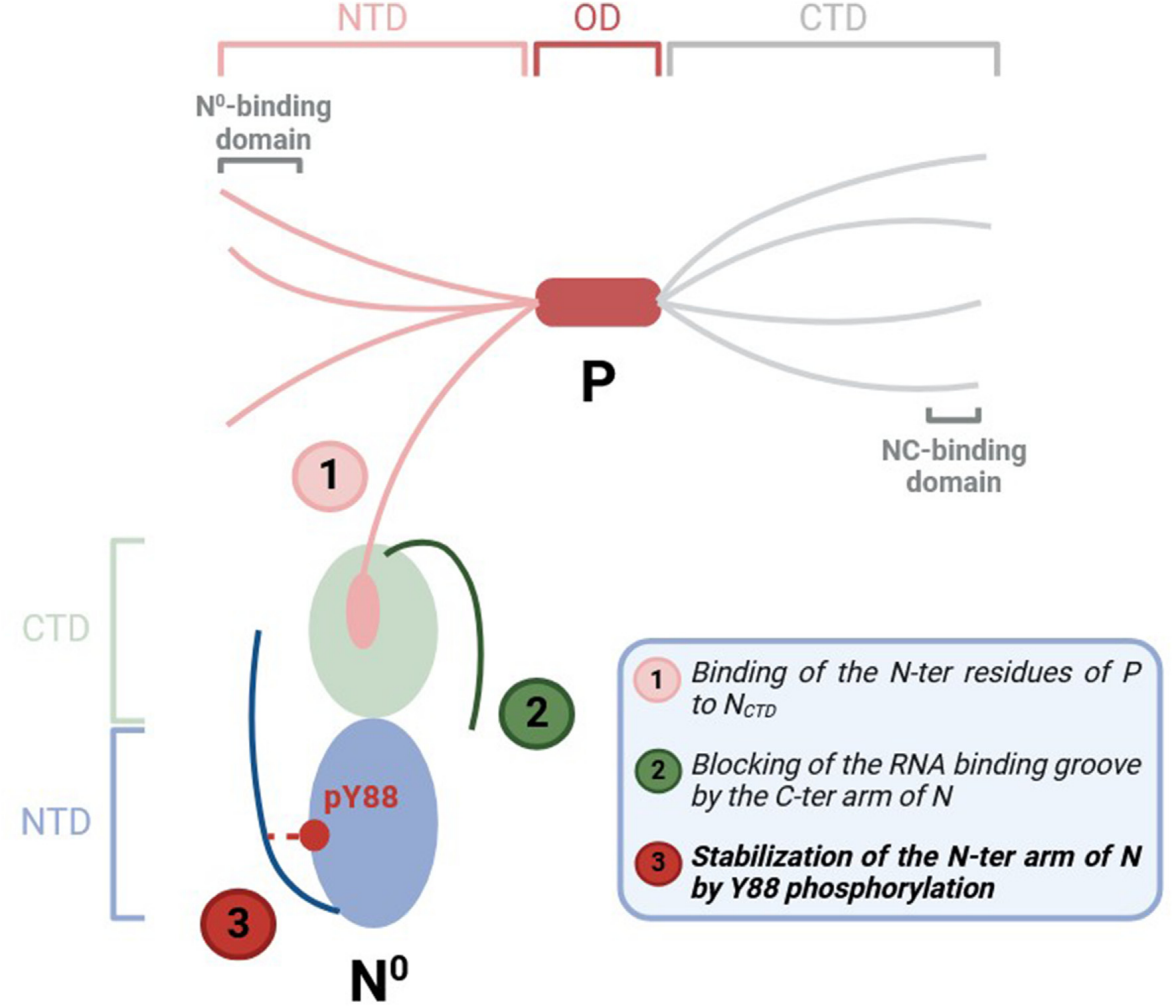
**Scheme of a P tetramer and monomeric N**^**0**^**protein, showing the “three clefts” involved in the stabilization of the N**^**0**^. CTD, C-terminal domain; NTD, N-terminal domain; OD, oligomerization domain.

As for all the members of the *MNV*s order, the spatial and temporal regulation of viral genome encapsidation is critical for efficient RSV replication. The study of the mechanisms sustaining the control and specificity of viral genome encapsidation remain challenging due to the strong tendency of N to nonspecifically interact with RNA and to concomitantly oligomerize when expressed alone. Although the P protein plays a key role of chaperone by interacting with neosynthesized N^0^, maintaining it monomeric and RNA free (31), the mechanisms involved in the switch from N^0^-P to N-RNA remain unknown. Noteworthy, although several *MNV*s N^0^-P complex structures have been solved, the purification of most of these complexes required the deletion of the N- and/or C-terminal arms of N (35, 36, 37, 38, 40, 50), suggesting the potential existence of specific mechanisms controlling these flexible domains to limit N oligomerization. This hypothesis is supported by the structure of HMPV N^0^-P complex, which was obtained by purification of a fusion protein consisting in the N terminal residues of P fused to the C terminus of full-length N (39). Interestingly it revealed a “two clefts” mechanism, with the P binding on the N_CDT_ expected to mainly impair N oligomerization, and a conformational rearrangement of the flexible C terminal arm of N which folds at the surface of the RNA binding groove thus blocking its accessibility (39). Although the structure of RSV N^0^-P is not solved yet, we have shown that this mechanism is conserved for RSV, using biochemical and biophysical characterization of copurified N deleted from its 30 N terminal residues with the 29 N terminal residues of P (41) (Fig. 7). This complex was shown to be stable up to 12 mg/ml, but did not allow to obtain crystals. More recently, we used a strategy similar to the one used for HMPV N^0^-P complex, and purified from *E. coli* a recombinant monomeric N surrogate composed of the full-length N and the 40 N terminal residues of P (N-P40) (45). However, this protein was prone to oligomerize upon concentration in the absence of RNA. Altogether, these results further sustain the fact that additional mechanisms could be required to stabilize the monomeric N.

PTMs are a widespread strategy used by both prokaryotic and eukaryotic cells to quickly and transiently modify proteins folding and/or activity (51, 52, 53, 54). Several PTMs, mostly phosphorylation, have been identified on RSV N, P, and M2-1 proteins (49, 55, 56, 57, 58, 59). While the role of P phosphorylation during replication and transcription steps still remains unclear (60, 61), phosphorylation of N and M2-1 were shown to be critical for the polymerase functioning (34, 49). The cyclic phosphorylation of M2-1, involved in the regulation of M2-1 interaction with P and mRNA, was also shown to depend on its interaction with P, which itself interacts with the phosphatase protein phosphatase 1 responsible for M2-1 dephosphorylation (34). These observations highlighted the complexity of the regulation of the activity of these viral proteins. Concerning the N protein, Asenjo *et al.* identified several phosphorylated tyrosine residues, and showed that Y38 phosphorylation affects viral RNA synthesis (49). However, the role of those PTMs in either the N^0^-P complex formation, the switch from N^0^-P to NCs assembly, NC-P or N-RNA interactions, or in the interaction with other partners of N was not clearly addressed.

Here, we specifically investigated the potential role of PTMs in the stabilization of N monomer. We first decided to produce N-P40 in insect cells. This protocol did not allow to improve the proportion of purified monomers, compared to N-P40 purified from *E. coli* (45). Nevertheless, mass spectrometry of this recombinant monomeric protein allowed to identify few peptides with PTMs, but only four with sufficient confidence: three phosphorylation on residues Y88, S153, and S276, and a di-methylation of residue K379 (Table 1). Noteworthy, these PTMs, which are known to be reversible and labile (62), were detected on the protein produced and purified in the absence of specific phosphatases or methylases inhibitors. Based on their respective localization at the N surface (Fig. 1), all these PTMs could impact either the interaction of N with P (or other partners) or N folding. We decided to focus on the residue Y88, which was also found phosphorylated on recombinant N-P40 produced in *E. coli*, but also importantly is involved in a key interaction between N protomers and is conserved between *Orthopneumoviridae* (18). These observations indeed strongly suggested that phosphorylation of Y88 could participate in the control of the N terminal arm of N, by limiting protomers interaction. We then assessed the role of this PTM by studying the impact of Y88D mutation, mimicking Y88 phosphorylation. We showed that Y88D mutation inhibited the polymerase activity, impacted the size of pseudo-IBs and seemed to increase the solubility of the protein (Fig. 2). Noteworthy, the study of Y88F mutant showed that this mutation also strongly affected the polymerase activity, but most probably by inducing aberrant oligomerization of N, although this hypothesis would have to be confirmed. We then compared the stability of N-P40 monomer upon production in *E. coli* and purification of WT or Y88D mutant in the absence or the presence of phosphatase inhibitors. Our results clearly showed that addition of phosphatase inhibitors improved the purification of WT monomeric N-P40, confirming the importance of phosphorylation for monomer stabilization (Fig. 3). Of note, Western blot analysis of purified N-P40 using anti-phosphotyrosine antibody suggested the presence of different phosphorylated tyrosine residues, in agreement with the results obtained by Asenjo *et al.* (49). Further characterization of these PTMs by LC-MS/MS would be required to confirm these results. More interestingly, Y88D mutation strongly improved the capacity to purify monomeric N-P40, and addition of phosphatase inhibitors did not further increase the proportion of monomers. However, the Y88D mutant protein still presented a tendency to aggregate upon concentration, which could suggest that this PTM, together with P binding and the rearrangement of the C-arm of N, may not be sufficient to completely stabilize the N^0^. We also showed that this mutation prevented RNA binding and affected pseudo-IBs formation *in vitro* (Fig. 4), but also in cells (Fig. 5). Altogether these results correlated with the defect of pseudo-IBs formation and the increased solubility of N observed upon expression of N Y88D mutant in eukaryotic cells (Fig. 2), and highlighted the pivotal role of Y88 phosphorylation in the stabilization of monomeric N protein. Finally, as the mutation Y88D can mimic but not reproduce a real phosphorylation, we further assessed the impact of Y88 phosphorylation by leading MD simulations on both monomeric and oligomeric N. Results of these simulations showed that Y88 phosphorylation should induce an intramolecular interaction between phosphorylated Y88 residue N and the residue R27 of N, stabilizing the conformation of N monomer (Fig. 6). In parallel, this phosphorylation would impair the critical interaction between R27 and the residues from the neighboring protomer within N-RNA oligomers.

In conclusion, although phosphorylation of the N protein of several *MNV*s including RSV has already been shown to play a role in viral polymerase functioning (63, 64, 65, 66, 67), the present study is the first one that allows to identify and characterize the role of N phosphorylation in the stabilization of N monomers, which could be considered as a third cleft involved in the regulation of N^0^ pool (Fig. 7). Such a mechanism of control of nucleoprotein oligomerization was also described for the nucleoprotein for Influenza virus, which genome is a segmented negative sense RNA (68, 69). In that case, phosphorylation of three serine residues was shown to improve monomer stability. Nevertheless, further investigation remains necessary not only to identify kinases and phosphatases involved in the regulation of Y88 phosphorylation of RSV N, but also to characterize the role of other PTMs of N. Given the critical role of transient protein-protein interactions for viral polymerase functioning, characterization of such PTMs and of the cellular enzymes responsible for these modifications would upscale the knowledge on *MNV*s polymerase functioning, but also open the way for new and specific antiviral strategies.

## Experimental procedures

### Cells

BHK-21 cells (clone BSRT7/5) constitutively expressing the T7 RNA polymerase (70) were grown in Dulbecco’s modified Eagle’s medium (Eurobio Scientific) supplemented with 10% fetal calf serum, 2 mM glutamine, and antibiotics. HEp-2 cells were grown in Modified Eagle’s medium (Eurobio Scientific), also supplemented with 10% fetal calf serum, 2 mM glutamine, and antibiotics. Cells were transfected using Lipofectamine 2000 (Invitrogen) as described by the manufacturer.

### Plasmids and bacmids

The pFastBac Dual (pFBD) vector with a synthetic sequence optimized for eucaryotic production of the N-P40 protein cloned into BamHI and SalI restriction sites was generated by GeneCust. This sequence was previously described (45). Briefly, it encodes for full-length N fused to the first 40 residues of P, with a N-terminal 6xHis tag and a TEV cleavage site upstream the N sequence. This plasmid was used for bacmid generation and baculovirus production. For bacmid generation, DH10EmBacY cells were transformed by the pFBD N-P40 plasmid. After overnight incubation at 37 °C, plates were stored at 4 °C for 2 to 3 days, and the colonies that remained white were selected for bacmid obtention. The sequence of N-P40 without any tag was also amplified by PCR from the pFastBac vector to be cloned in the pEGFP-C1 vector, into the SalI and BamHI restriction sites. This plasmid was used for HEp-2 cells transfection either alone or with pcDNA-P and pcDNA-N plasmids (71). Other plasmids for eukaryotic expression of the RSV N, P, M2-1, and L proteins, designated pN, pP, pM2-1, and pL, and the pM/Luc subgenomic minigenome which encodes the firefly luciferase (Luc) reporter gene under the control of the M/SH gene start sequence have been described previously (24, 72). For production of recombinant proteins in *E. coli*, the previously described pET-N, pET-N-P40, and pGEX-PCT plasmids were used (20, 45). Mutations were generated using the Q5 Site-Directed Mutagenesis Kit (New England Biolabs), following the manufacturer’s instructions. Sequence analysis was carried out to check the integrity of all the constructs.

### Antibodies

The following primary antibodies were used for immunofluorescence and/or immunoblotting: a rabbit anti-N antiserum (73), a rabbit anti-P antiserum (72), a mouse monoclonal anti-P (74), a mouse monoclonal anti-phosphotyrosine (Santa Cruz Biotechnology), and a mouse monoclonal anti-β-tubulin (Sigma-Aldrich). Secondary antibodies directed against mouse and rabbit IgG coupled to HRP (SeraCare) were used for immunoblotting, and antibodies directed against mouse and rabbit IgG coupled to Alexa 594 or Alexa 488 (Invitrogen) were used for immunostaining.

### Production and purification of recombinant proteins

For production of N-P40 recombinant protein in *E. coli*, BL21 (DE3) bacteria were transformed with the pET-N-P40 plasmids. Cultures were grown at 37 °C in 2xYT medium containing 50 μg/ml kanamycin. After 8 h, the same volume of 2xYT was added to the cultures, and protein expression was induced by adding 80 μg/ml IPTG overnight at 28 °C. Bacteria were harvested by centrifugation, and pellets resuspended in lysis buffer (20 mM Tris–HCl, pH 8.5, 500 mM NaCl, 0.1% Triton X-100, 10 mM imidazole, 1 mg/ml lysozyme, and complete protease and phosphatase inhibitors (Roche)). After incubation on ice for 30 min, the lysates were sonicated, RNase A was added (final concentration of 1 U/ml), and NaCl was added, up to a concentration of 1M. After centrifugation at 10,000g for 30 min at 4 °C, the lysates were incubated with Chelating Sepharose Fast Flow beads (GE HealthCare) charged with Ni2+, for 1 h at 4 °C. Beads were washed with washing buffers (20 mM Tris–HCl, pH8.5, 1M NaCl) with increasing imidazole concentrations (10, 50, and 100 mM), before elution of the protein using 800 mM imidazole. Purified proteins were loaded on a Hi-Load 16/600 Superdex 200 column (GE HealthCare) and eluted in 20 mM Tris–HCl, pH 8.5, 1M NaCl. Then, the purified proteins were dialyzed against 20 mM Tris–HCl, pH 8.5, with decreasing NaCl concentrations (500 and 300 mM) buffers, for 4 h each, at 4 °C. Proteins were then concentrated using a centrifugal concentrator with a molecular weight cut-off of 10 kDa (Vivaspin turbo 4, Sartorius).

For eukaryotic production of N-P40 recombinant protein, High-Five cells (Thermo Fisher Scientific, catalog number B85502) were infected at multiplicity of infection 2 with the baculovirus encoding an optimized sequence of N-P40 construction, for 72 h. Cells were centrifuged at 3000g for 5 min and resuspended in 15 ml of lysis buffer (20 mM Tris–HCl, pH 8.5, 500 mM NaCl, 0.1% Triton X-100, 10 mM imidazole, 1 mg/ml lysozyme, and complete protease and phosphatase inhibitors). Purification was then performed following the protocol described above.

### Mass spectrometry analysis

Samples were loaded on a SDS-PAGE gel (NuPAGE 4–12% Bis Tris gel, Invitrogen), and each strip of interest was cut and washed successively for 15 min with a solution of (i) 10% acetic acid and 40% ethanol (ii) acetonitrile and 50 mM ammonium bicarbonate mixture (1:1). Then, disulfide bonds were reduced with DTT 10 mM at 56 °C during 30 min and alkylated with iodoacetamide 55 mM during 45 min in the dark at room temperature. Digestion was performed in 50 mM ammonium bicarbonate (pH 8.0) with a protein:enzyme ratio of 1:100 (trypsin, AspN, and chymotrypsin), overnight at 25 °C for trypsin, AspN, and chymotrypsin. Peptides were extracted by adding 0.5% TFA/50% ACN solution, the supernatant was lyophilized and stored at −20 °C. Lyophilized peptides were then resuspended in 20 to 40 μl of 2% ACN and 0.08% TFA buffer and MS analyses were performed on a Dionex U3000 RSLC coupled to an Orbitrap Fusion Lumos Tribrid mass spectrometer (Thermo Fisher Scientific) at the Plateforme d'Analyse Protéomique de Paris Sud Ouest (PAPPSO), Jouy-en-Josas, France (http://pappso.inrae.fr/).

The tryptic peptides were loaded on a PepMap Neo trap column (300 μm i.d. x 5 mm, with a particle size of 5 μm, 100A, Thermo Fisher Scientific) and separated using a C18 column (50 cm × 75 μm i.d.2 μm particle size). The peptides were eluted on the nLC system through the following gradient elution program: 2.5 to 35% buffer B (80% ACN and 0.1% TFA) within 0 to 50 min, 35 to 45% buffer B in 50-55 min, 45 to 98% buffer B in 55 to 57 min. The detection of peptides was acquired in the data-dependent acquisition mode, for MS1 signals, the electrospray voltage was set at 1600V, in positive mode. MS scans were performed at 120,000 resolution, m/z range 400‒2000 Da. LC-MS/MS analysis was performed in a data-dependent acquisition, with a top speed cycle of 2.5 s for the most intense double or multiple charged precursor ions. Ions in each MS scan over threshold 20,000 were selected for fragmentation (MS2) by higher energy collisional dissociation at 30% for identification and detection in the orbitrap followed by a top speed MS2 fragment ions. Precursors were isolated in the quadrupole with a 1.6 m/z window and dynamic exclusion within 10 ppm during 60 s was used for m/z-values already selected for fragmentation. The AGC targets are fixed as standard for MS and MS/MS analysis. Polysiloxane ions m/z 445.12002, 519.13882 and 593.15761 were used for internal calibration.

### Protein identification from LC-MS/MS

Protein identification was performed using Peaks Studio (https://www.bioinfor.com/peaks-studio/) 10.6 software. A fasta format database was used including the genomes of uniprotkb_Trichoplusia_Ni (20,889 entries) or *Homo sapiens* (20,403 entries), the N_RSV protein and the common contaminants proteins. We used PTMs parameters, focusing on phosphorylation and methylations modifications but oxidation of methionine and acetylation were also selected as potential modification. Filter parameters were set at 10 ppm for parent mass error tolerance, 0.02 Da for fragment mass error tolerance and a maximum of three missed cleavages were fixed.

### Minigenome assay

Cells at 90% confluence in 96-well dishes were transfected with a plasmid mixture containing 62.5 ng of pM/Luc, 62.5 ng of pN, 62.5 ng of pP, 31.25 ng of pL, and 15.5 ng of pM2-1 as well as 15.5 ng of pRSV-β-gal (Promega) to normalize transfection efficiencies (24). Transfections were done in triplicate, and each independent experiment was performed three times. Cells were lysed 24 h after transfection in luciferase lysis buffer (30 mM Tris, pH 7.9, 10 mM MgCl2, 1 mM DTT, 1% Triton X-100, and 15% glycerol). The luciferase activities were determined for each cell lysate with an Infinite 200 Pro (Tecan) and normalized based on β-galactosidase (β-gal) expression.

### Fluorescence microscopy

Cells grown on coverslips were transfected with pN (WT or mutant) and pP for BSRT7/5 cells or pEGFP-N-P40, pcDNA P, and pcDNA-N for HEp-2 cells. Twenty-four hours after transfection, cells were fixed with 4% paraformaldehyde for 20 min. Fixed cells were permeabilized, blocked for 30 min with PBS containing 0.1% Triton X-100 and 3% bovine serum albumin, and then successively incubated for 1 h at room temperature with primary and secondary antibody mixtures diluted in PBS containing 3% bovine serum albumin. For labeling nuclei, Hoechst 33342 (Invitrogen) was added during incubation with the secondary antibodies. Coverslips were mounted in ProLong gold antifade reagent (Invitrogen). Cells were observed with a Nikon TE200 microscope equipped with a CoolSNAP ES^2^ (Photometrics) camera, and images were processed using MetaVue (Molecular Devices; https://www.moleculardevices.com/products/cellular-imaging-systems/high-content-analysis/metamorph-microscopy/) and ImageJ software (https://imagej.net/downloads).

### SDS-PAGE and Western blots

Protein samples were separated by electrophoresis on 12% polyacrylamide gels in Tris–glycine buffer. All samples were boiled for 3 min prior to electrophoresis. For Western blot, proteins were then transferred to a nitrocellulose membrane (Bio-Rad). The blots were blocked with 5% nonfat milk in PBS Tween 20 0.2%, followed by incubation with primary, then secondary antibodies. Western blots were developed using Clarity Western ECL substrate (Bio-Rad) and exposed using the Bio-Rad ChemiDoc Touch Imaging System (Bio-Rad).

### Band shift on native agarose gel

Subsequently, 50% sucrose loading buffer was added to the samples before loading on native 1% agarose gel. Migration was performed in 1× Tris–glycine buffer during 2 h at 80V, before staining with amido black 10B.

### Solubility assay

Cells plated in 24 well-plates were washed 24 h post transfection with PBS, and resuspended in the presence of PBS-EDTA 2.5 mM. After 5 min centrifugation at 2000 rpm, cells present in the pellet were resuspended in 120 μl lysis buffer (50 mM Tris–HCl, pH 7.4, 2 mM EDTA, 150 mM NaCl, and 1% NP-40), and sonicated in a bath with a 10 s pulse. After centrifugation at 13,000 rpm at 4 °C for 20 min, the supernatant containing soluble proteins and the pellets were recovered and mixed with Laemmli buffer. Samples were boiled and proteins were resolved by SDS-PAGE.

### *In vitro* reconstitution of IBs

As previously described (44), P-BFB and 14-mer RNAs (5′-ACGCGAAAAAAUGC-3′) were incubated alone or in the presence of monomeric N-P40 recombinant proteins in 20 mM Tris–HCl pH 8.5, 150 mM NaCl buffer on glass slides, and the molecular-crowding agent Ficoll was added on the droplets of solution. Coverslips were then laid on the droplets. The formation of droplets was observed with a Nikon TE200 inverted microscope equipped with a Photometrics CoolSNAP ES^2^ camera. Images were processed using MetaVue (Molecular Devices) and ImageJ software.

### Molecular dynamics simulations

Classical MD simulations were used to study the impact of Tyr88 phosphorylation on the dynamics of N-N interactions. Monomeric and dimeric N models were extracted from a 2.8 Å resolution cryo- EM structure of HRSV N-RNA rings (Protein Data Bank code 80P2) (17). MD systems corresponding to both unphosphorylated and phosphorylated monomers and dimers were set up using the solution builder tool from CHARMM-GUI input generator (75). Briefly, each system was solvated in a rectangular periodic box, which size was determined by the biomolecular extent, resulting in the addition of approximately 48k and 65k TIP3P for monomers and dimers, respectively. The systems were neutralized by adding 150 mM NaCl. Each four systems were then energy minimized, equilibrated in NVT ensemble and simulated for ∼ 200 ns in three independent trajectories in GROMACS2024 (76) using CHARMM-GUI scripts (77). The CHARMM36m force field was used for all simulations (78). The trajectories were analyzed using GROMACS tools to extract interresidue distances, as well as RMSDs and number of hydrogen bonds as a function of simulation time.

## Data availability

The data described in the manuscript are contained within the manuscript and the supplementary files.

## Supporting information

This article contains supporting information.

## Conflict of interest

The authors declare that they have no conflicts of interest with the contents of this article.
