# Supplementary material for: Regulation of respiratory syncytial virus nucleoprotein oligomerization by phosphorylation
Source: J Biol Chem. 2025 Feb 3;301(3):108256. doi: 10.1016/j.jbc.2025.108256 (PMC11910103; doi:10.1016/j.jbc.2025.108256)
Supplement: Supplemental Figure [file mmc1.docx]

**
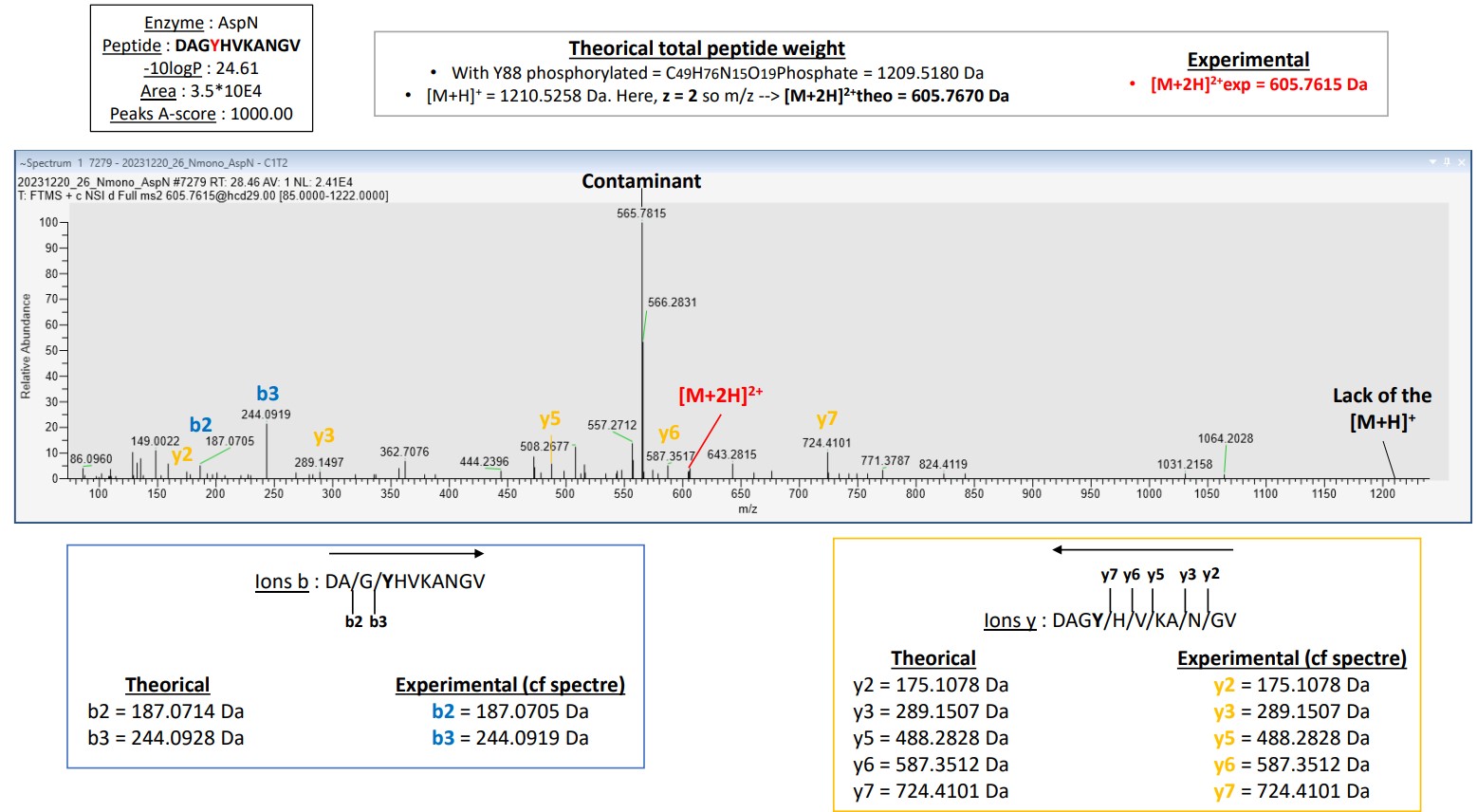
**

**Fig Supp 1**

Purified recombinant N-P40 protein from both P1 and P2 peaks was separately digested by AspN. The DAGYHVKANGV peptide was among the products specifically detected from the monomeric fraction digestion (peak 2). The theorical molecular weight of this un-modified peptide is 1129.548 Da (C_49_H_76_N_15_O_19_). On the experimental peptide, the measured [M+2H]^2+^ is 605.7615 Da, with z = 2, meaning [M+H]^+^ = 1210.52 Da. Its experimental molecular weight is therefore 1209.52 Da. The peptide is cut on two ions b (blue) and five ions y (orange), in such a way that it pinpoints the observed mass difference to the tyrosine. The difference between theorical and experimental molecular weight is 79.97 Da, which corresponds to the exact weight of a phosphate group, indicating that this tyrosine is phosphorylated. The -10logP value for this peptide, which serves as a confidence index, is 24.61 which is relatively medium, and the Area is in the order of magnitude 10^4^, also medium. This data was also analyzed using the Peaks Software which provided us with its own confidence index, an A Score of 1000, which is very good. (The overall flattened appearance of most peaks from this specter is due to the presence of a contaminant with strong peaks at 565.7815 Da).
